# High-resolution DNA copy number and gene expression analyses distinguish chromophobe renal cell carcinomas and renal oncocytomas

**DOI:** 10.1186/1471-2407-9-152

**Published:** 2009-05-18

**Authors:** Maria V Yusenko, Roland P Kuiper, Tamas Boethe, Börje Ljungberg, Ad Geurts van Kessel, Gyula Kovacs

**Affiliations:** 1 Laboratory of Molecular Oncology, Medical Faculty, Ruprecht-Karls-University, Heidelberg, Germany; 2 Department of Human Genetics, Radboud University Nijmegen Medical Centre, Nijmegen, the Netherlands; 3 Department of Urology, University of Pécs Medical School, Pécs, Hungary; 4 Department of Urology, Umeå University, Umeå, Sweden

## Abstract

**Background:**

The diagnosis of benign renal oncocytomas (RO) and chromophobe renal cell carcinomas (RCC) based on their morphology remains uncertain in several cases.

**Methods:**

We have applied Affymetrix GeneChip Mapping 250 K NspI high-density oligoarrays to identify small genomic alterations, which may occur beyond the specific losses of entire chromosomes, and also Affymetrix GeneChip HG-U133 Plus2.0 oligoarrays for gene expression profiling.

**Results:**

By analysing of DNA extracted from 30 chRCCs and 42 ROs, we have confirmed the high specificity of monosomies of chromosomes 1, 2, 6, 10, 13, 17 and 21 in 70–93% of the chRCCs, while ROs displayed loss of chromosome 1 and 14 in 24% and 5% of the cases, respectively. We demonstrated that chromosomal gene expression biases might correlate with chromosomal abnormalities found in chromophobe RCCs and ROs. The vast majority genes downregulated in chromophobe RCC were mapped to chromosomes 2, 6, 10, 13 and 17. However, most of the genes overexpressed in chromophobe RCCs were located to chromosomes without any copy number changes indicating a transcriptional regulation as a main event.

**Conclusion:**

The SNP-array analysis failed to detect recurrent small deletions, which may mark loci of genes involved in the tumor development. However, we have identified loss of chromosome 2, 10, 13, 17 and 21 as discriminating alteration between chromophobe RCCs and ROs. Therefore, detection of these chromosomal changes can be used for the accurate diagnosis in routine histology.

## Background

Renal oncocytomas (RO) and chromophobe renal cell carcinomas (RCC) make up approximately 10% of renal cell tumors (RCT). Although chromophobe RCC has a better prognosis than conventional or papillary RCC, it is a malignant tumor with a tendency to sarcomatoid transformation and metastatic growth in around 10% of the cases [1-4]. Renal oncocytoma, in spite of its growth into small veins or "infiltration" to the parenchyma or perinephric fatty tissue, is a benign tumor [5]. Taking into account the biology of the two types of neoplasms, the differential diagnosis is of clinical importance.

We have detected complex losses of chromosomes 1, 2, 6, 10, 13, 17 and 21 in 70%–90% of the chromophobe RCCs by karyotyping, chromosomal CGH and microsatellite analysis [6-9]. Recently, the specificity of these chromosomal changes has been confirmed by other investigators [10]. Loss of chromosomes 1, 14 and the Y chromosome or translocation between chromosome 11q13 and other chromosomes or random genetic changes have been described in ROs [11,12]. The lack of genetic changes specific for other types of RCTs combined with the histological characteristics may also be helpful in the diagnosis of RO [13,14]. The complex genetic alterations occurring in conventional, chromophobe or papillary RCCs can also be used for differential diagnosis of "unclassified" RCTs by karyotyping, microsatellites and BAC-array technologies [15-17].

The resolution of karyotyping and chromosomal CGH is limited by DNA alterations of approximately 5–10 Mb. These techniques revealed the loss of entire chromosomes or chromosomal arms making it impossible to localize putative tumor suppressor genes. Global gene expression studies suggested that genes mapped to chromosomes displaying monosomie in chromophobe RCCs are generally down-regulated but no specific genes have been selected and confirmed at the protein level [18]. Other studies analysing the global gene expression profiling showed that several hundreds of genes are over-expressed in both chromophobe RCCs and renal oncocytomas irrespectively of their chromosomal localization and down-regulated in other types of renal cell tumors [19-21]. However, immunohistochemical studies of selected genes did not confirm the high specificity gene expression data [22,23].

Recent development in the array technology enables the detection of small DNA copy number changes throughout the entire genome, which may mark the locus of putative tumor genes. To detect such regions, we have analysed 30 chRCCs and 42 ROs using high-density SNP-based oligoarrays. We have also applied gene expression profiling to examine the molecular signature in a series of RCTs including chromophobe RCCs and ROs. The data obtained from both sources were combined and a consistent relationship between underexpression of genes located on chromosomes, which are lost from the genome of chRCC, was detectable.

## Methods

### Tumor samples

Fresh tumor and corresponding normal parenchymal tissues were obtained by nephrectomy at the Departments of Urology, Ruprecht-Karls-University Heidelberg, Germany, University of Pecs, Hungary and University of Umea, Sweden from 30 chromophobe RCCs and 42 ROs. One part of the tumor and normal kidney tissues was immediately snap-frozen in liquid nitrogen and stored at -80°C whereas the remaining tumors with the nephrectomy specimen was fixed in 4 per cent buffered formalin and processed for histological examination. The histological diagnosis according to the Heidelberg Classification of Renal Cell Tumours was established by one of the authors [24]. The collection and use of tissue samples for this study was approved by the Ethics Committee of the University of Heidelberg.

### DNA and RNA extraction

A frozen tumor sample was placed in a plastic Petri dish, covered with 1 ml TE9 buffer (0.5 M Tris-HCl, pH 9.0; 0.1 M EDTA), and allowed to thaw. The tumor cells were then carefully scraped or pushed out to separate them from stromal tissue under an inverted microscope by a pathologist (GK) experienced in this technique. The stromal rests were discarded. By this method contamination by normal cells was reduced to a minimum as it was demonstrated by previous microsatellite analysis [13]. Tumor cells were resuspended in 5 ml TE9 buffer with 1% SDS and 0,2 mg/ml proteinase K and incubated for 3 hours at 55°C. DNA was extracted by phenol-chloroform and dissolved in TE (10 mM Tris-HCl, pH 8.0; 1 mM EDTA) buffer after ethanol precipitation. Frozen tissue samples from chRCCs and ROs, other types of renal cell tumors as well as of adult normal kidneys were homogenized in TRIzol reagent (Invitrogen), and high-quality total RNA was extracted according to the manufacturer's recommendations. The quality was assessed using the ratio of absorbance at 260 nm to 280 nm (A_260_/A_280_) and by running on denaturing 1% agarose gel to confirm the presence of non-degraded RNA.

### SNP-array study and data analysis

The oligonucleotide array experiments were performed using the Affymetrix GeneChip Mapping 250 K NspI arrays (Affymetrix Inc., Santa Clara, CA, USA). Male and female reference DNA pools containing equal amounts of genomic DNA from 10 healthy donors were used for normalisation purposes.

DNA labeling, hybridization, washing and staining of the 250 K NspI arrays were performed according to the manufacturer's instructions. First, 250 ng of genomic DNA was digested with NspI and then ligated to an adaptor with T4 ligase. A generic primer recognizing the adapter sequence was used to amplify adapter ligated DNA fragments with PCR conditions optimized to amplify preferentially fragment in the range of 200 to 1.100 bp. To obtain enough PCR product, three 100 μl PCRs were set up for each adaptor ligated DNA sample. The PCR products from the three reactions were then pooled and purified with the DNA amplification clean up kit (Clontech Laboratories, Mountain View, CA, USA). A final 90 μg PCR product was fragmented with DNaseI and a sample of the fragmented product was visualized on a 2% agarose gel to confirm that the average size was smaller than 180 bp. Fragmented PCR products were then end labeled with biotin, denatured and hybridized to the arrays for 18 hrs. After hybridization the arrays were washed and stained using an Affymetrix GeneChip Fluidic Station 450 and scanned by the GeneChip Scanner 3000 7 G.

Mapping information for SNP, RefSeq and Cytoband locations were determined based on Affymetrix annotations and HG17 build of the genome sequence (May 2004) from http://genome.ucsc.edu/. The allelic intensity of each SNPs from the GeneChip Operating Software was measured using the GeneChip Genotyping analysis software (GTYPE v4.0). Copy number intensities were calculated using the public domain software package CNAG (Copy Number Analyser for GeneChip), version 2.0, and detected using the implemented Hidden Markow Model as well as by visual inspection [25]. To distinguish between tumor-associated copy number changes and naturally occurring copy number variation (CNV), all changes were compared with public databases containing normal copy number variants occurring in the general population http://projects.tcag.ca/variation/ as well as with a in-house database of 250 unrelated healthy individuals. Regions of overlap after excluding CNVs were calculated using a standard Microsoft Excel software package. To obtain genome-wide view of chromosomal imbalance in chRCCs and ROs the output files (.TXT) from CNAG 2.0 were converted into the format suitable for processing in WEB-interface GWA http://bioinformatics.cancerresearchuk.org/cazier01/GWA_Events.html.

### Gene expression profiling and data analysis

Gene expression profiling in 66 samples (26 conventional RCCs, 17 papillary RCCs, four chromophobe RCCs, four ROs, two collecting duct carcinomas, one mucinous and spindle cell tumor, four Wilms' tumors, one clear cell sarcoma of the kidney, one rhabdoid tumor of the kidney as well as four adult and two fetal normal kidneys) was obtained using HG-U133 Plus2.0 GeneChip oligonucleotide microarray (Affymetrix Inc.; see manufacturer's manual for detailed protocol) containing 54,675 probe sets that correspond to 38,500 genes (and > 47,400 transcripts). Total RNA was purified with Qiagen RNeasy Mini Kit (Qiagen), and the cRNA synthesis and hybridization was performed by the Genomics Core Facility of EMBL (Heidelberg, Germany). The stained arrays were scanned, and perfect match and mismatch features on the scanned microarray images were quantified using default settings in Microarray Suite 5.0 software (MAS 5.0, Affymetrix Inc.) yielding signal intensity for each probe on the array. The hybridization (raw) data have been deposited in NCBI's Gene Expression Omnibus repository http://www.ncbi.nih.gov/geo/ and are available under the accession number "GSE11151".

The robust multi-array average algorithm of R and RMA implementation in Bioconductor package http://www.bioconductor.org was used to perform preprocessing of the .CEL files, including background adjustment, quartile normalization, and summarization. Expression measurements were transformed by computing the base-two logarithm before further analysis. Relative expression profiles were generated from the individual tumor expression profiles and the mean expression values of the four individual normal adult kidney expression profiles.

### Quantative real-time PCR

Two μg of total RNA was reverse transcribed with SuperScript II Reverse Transcriptase (Invitrogen) in 25 μl reaction volume. Six μl of 1:16 diluted cDNA was amplified with 0.5 μM of each forward and reverse primer and 7.5 μl of the Platinum SYBR Green qPCR SuperMix UDG kit (Invitrogen) in 15 μl final volume. The PCR was performed in the Opticon Real Time PCR Machine (MJ Research Inc., Watertown, MA.). Primer sequences and PCR conditions used in this study are available upon request. Specificity of the PCR products was verified by analysis of melting curves generated at the end of the cycles. For relative quantification, standard curves were performed from a 5-step dilution series of pooled normal kidney cDNA for both gene specific and also GAPDH and ACTB reactions. The relative expression level was calculated by dividing the gene specific expression with the parallel GAPDH and ACTB expression.

## Results

### Copy number changes in chromophobe RCCs

**Figure 1:**
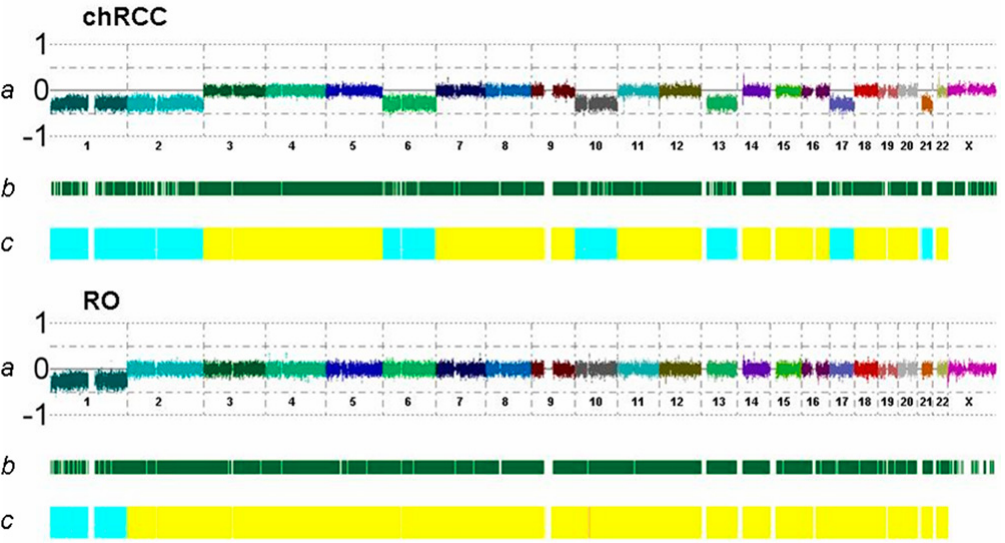
**Copy number alteration in a chromophobe RCC and RO**. (A) Representative genome view of copy number alterations of chromosomes 1, 2, 6, 10, 13, 17 and 21 in a chromophobe RCC and of chromosome 1 as a single genomic change in a RO. (B) The dark green bars represent heterozygous SNP calls in tumors. (C) The yellow bar marks the copy number data in a color-coded HMM model (pink: copy number 3, yellow: copy number 2, blue: copy number 1).

**Figure 2:**
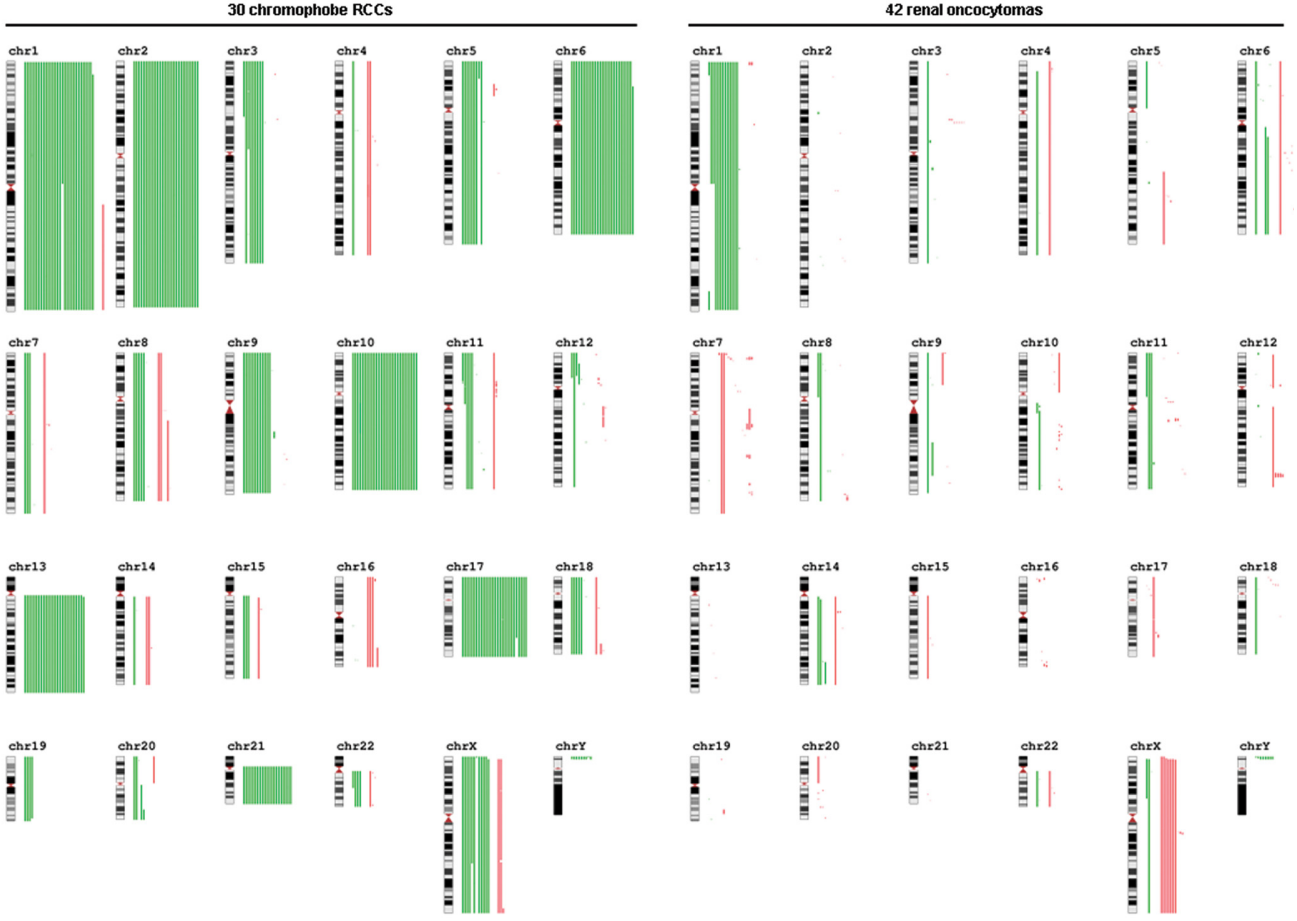
**Summary of genomic imbalances in 30 chRCCs and 42 ROs obtained with 250 K SNP array analysis**. Green lines on the right of ideograms indicate losses, whereas red lines represent gains. Notice the smaller overlapping deletions at chromosome 11p and 12p in two and three cases of chRCC, respectively. Notice the loss of chromosome 1 and gain of the X chromosome in RO. Loss at the PAR1 region indicates the loss of the Y chromosome.

**Figure 3:**
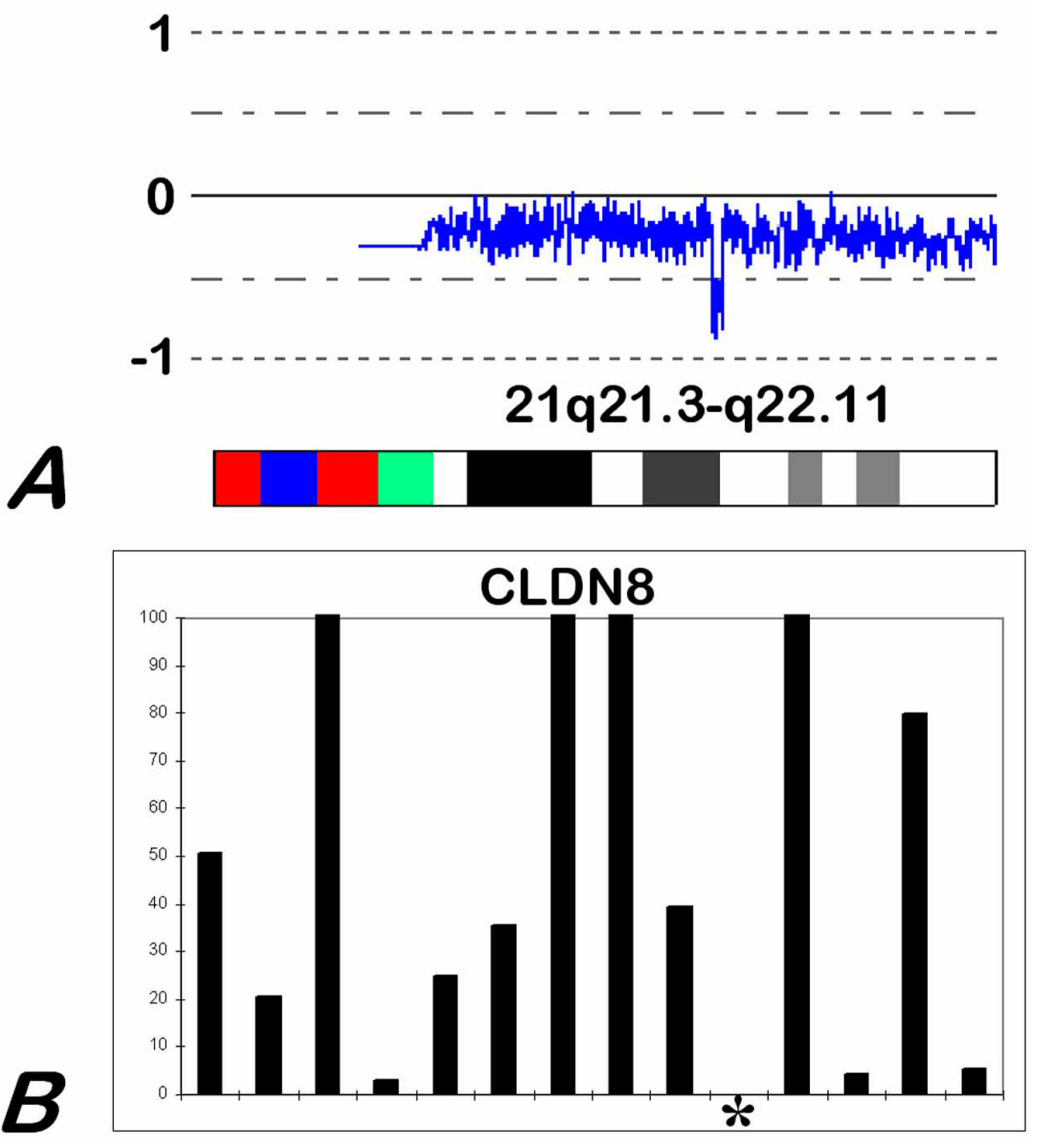
**Homozygous deletion and gene expression**. (A) A case of chromophobe RCC with a homozygous deletion at 21q21.3-q22.11 as revealed by SNP-based analysis. (B) Corresponding to the loss of both copies of the gene sequences the lack of expression of CLDN8 gene as obtained by quantitative real-time PCR.

In our series of chromophobe RCCs loss of the entire chromosome 1, 2, and 10 was the most frequently observed alteration followed by loss of chromosomes 17, 6, 13 and 21 (Figure 1 and Figure 2). In addition, we found a frequent loss of chromosome 9 (40%), 5 (27%), and 3 (23%). Evaluating the PAR1 on X and Y chromosomes indicated a loss of X and Y in 37% of the cases each. Some other chromosomal losses occurring in 3 to 15% of the cases may be classified as random genetic change. Taking all chromosomal losses into account, chromophobe RCCs in this series are characterised by loss of 5 to 13 chromosomes leading to low chromosome number. Several hemi- or homozygous deletions or gains of hundreds of kb to several Mb in size occurred at distinct chromosomal regions, each only in one or two cases (Table 1). We have analysed smaller deletions, which occurred at the chromosomes specifically involved in the genetics of chromophobe RCCs. A homozygous loss of 710 kb at chromosome 1p22.1 including five genes and another homozygous losses of 5.9 Mb at chromosome 2q22.3-q23.2 and 29.17 Mb at chromosome 10q11.23-q22.3 were seen in the same chromophobe RCC (Figure 3). In two other cases of chRCC, an approximately 950 kb homozygous deletion at chromosome 2q13 affecting 5 genes and a homozygous loss of approximately 600 kb at chromosome 21q21.3-q22.11 including 6 genes were revealed. A quantitative RT-PCR analysis of the genes BUB1 and CLDN8 in 19 chromophobe RCCs and 29 ROs did not revealed a correlation between copy number changes, e.g. loss of one allele and gene expression and (data not shown). The lack of expression of CLDN8 were seen only in the single case showing the homozygous deletion, which suggests the complete loss of gene sequences (Figure 3). Evaluating of the other genes (Table 1) from the small homozygous deletions on the Affymetrix panel has not showed a correlation between expression level and chromosomal loss. Large deletions involving a high number of genes were detected at chromosome 1q, 6, 17 and 21q whereas loss or gains at distinct loci occurred at chromosome 10.

**Table 1:** Gains and losses of DNA segments larger than 0.5 Mb in 30 chromophobe RCCs and 42 ROs.

Tumor	Chromosomal location	Size, Mb	G/L/HL*	Genes
chRCC	1p36.21-q44	232.12	L	
RO	1p36.33-p36.21	14.6	L	
chRCC	1p22.1	0.71	HL	MTF2, TMED5, NY-SAR-41, DR1, BCAR3
RO	1q42.2-q44	18.1	L	
chRCC	2q13	0.95	HL	BENE, NPHP1, LIMS3, RABP2L1, BUB1
chRCC	2q22.3-q23.2	5.9	HL	
RO	3p14.3	1.10	G	IL17RD, HESX1, APPL, FLJ44290, ARF4, SLMAP, FLNB
RO	3q13.11	2.72	L	ALCAM, CBLB
chRCC	5p14.1	1.54	G	CDH9
RO	5q21.3-q35.3	71.48	G	
chRCC	6p22.2-q27	145.86	L	
RO	6q13-q27	96.3	L	
RO	6q15	0.74	G	GABRR2, UBE2J1, RRAGD, ANKRD6, MDN1, CASP8AP2, OX62, BACH2
RO	6q24.2-q24.3	0.69	G	EPM2A, FBXO30, SHPRH
RO	7q11.22-q11.23	5.65	G	
RO	7q11.22-q11.23	3.30	G	
chRCC	7q11.22-q11.23	0.92	G	CALN1
RO	7q22.1	2.78	G	
RO	7q34	0.52	G	TIF1, Loc136306, ATP6V0A4
RO	9p24.3-p23	31.65	G	
RO	9q22.1-q33.2	32.75	L	
RO	10p15.3-p11.1	38.92	G	
chRCC	10q11.22	0.63	G	PPYR1, ANXA8
RO	10q11.23-q21.1	1.4	L	
RO	10q11.23-q21.1	10.2	L	
chRCC	10q11.23-q22.3	29.17	HL	
RO	10q21.1-q26.3	78.1	L	
RO	10q21.1-q26.3	78.1	L	
RO	10q22.3	1.71	G	
RO	11p15.2-p15.1	1.2	L	SOX6
RO	11p15.5-p12	43.10	L	
chRCC	11p15.5-p12	37,64	L	
RO	11p11.2	0.52	G	OR4X2, OR4X1, OR4S1, OR4C3, OR4A47
RO	11p11.2	0.40	G	
RO	11p11.2-p11.12	0.63	G	
RO	11q13.2	2.07	G	
RO	11q22.3-q23.1	2.18	L	
chRCC	12p13.33-p12.1	22.49	L	
chRCC	12p13.33-p12.1	24.61	L	
RO	12p13.33-p11.1	32.76	G	
chRCC	12p13.2-p11.21	20.49	L	
chRCC	12q13.13-q21.1	20.12	G	
RO	12q13.2-q24.33	79.05	G	
RO	14q31.3-q32.33	22,4	L	
chRCC	15q13.3	1.01	G	CHRNA7, ARGHAP11A
RO	16p13.3	1.57	G	
chRCC	17p13.3-q23.3	59.77	L	
chRCC	18q22.2-q23	10.32	G	
RO	20p13-p11.1	26.22	G	
chRCC	20q11.1-q11.22	4.30	L	
chRCC	20q13.2-q13.33	10.01	L	
chRCC	21q21.3-q22.11	0.59	HL	CLDN17, CLDN8, Loc138818, KRTAP13-1, KRTAP19-1, KRTAP19-3
chRCC	22q11.1-q12.3	16.5	L	
chRCC	Xp22.33-q22.3	105.26	L	
RO	Xq13.3-q21.1	1.76	G	

* G – gain, L – loss, HL – homozygous loss

### Copy number changes in renal oncocytomas

Loss of the entire chromosome 1 occurred in 33% of the ROs. The loss at the PAR1 region indicated the loss of the Y chromosome in 29% of the cases (Figure 2). We also found loss of the entire chromosome 14 in two cases. Loss of chromosomes 3, 6, 8, 9, 18, and 22 occurred each in one case. A smaller deletion of 14.6 Mb and 18,1 Mb was detected at chromosome 1p36.33-p36.21 and 1q42.13-q44 region, respectively. Whether these deletions mark genes involved in the development of ROs is not yet known. The smallest overlapping deletion occurring in two ROs at 11p15.2-p15.1 includes the SOX6, whereas the smallest overlapping gain at 11p11.2 in three ROs includes five members of the olfactory receptor proteins. A hemizygous loss of 22 Mb was seen at chromosome 14q31.3-q32.33 in one of the ROs, which may mark the loci of candidate genes. We have also detected a gain of signal at chromosome 3p14.2 in three ROs developed in the same kidney. The duplicated region corresponds to exon 5 of the FHIT gene at the most common fragile site FRA3B. Several other changes, each occurring in one case, are listed in Table 1. Of interest, gains at distinct chromosomes were more frequent in ROs than loss of DNA sequences.

### Common and discriminating DNA alterations of diagnostic importance

Some of the chromosomal changes occurred in both type of tumors albeit at different frequency (Figure 2). Loss of chromosome 1p occurred in 23% of the ROs and 93% of chromophobe RCCs. Whether the same genes are affected by the copy number alterations in chromophobe RCCs and ROs remains to be cleared. Loss of the entire chromosome 6 was seen in 88% of chromophobe RCCs, whereas two ROs showed loss of the long arm of chromosome 6. Overlapping alterations at chromosome 10 in one chRCC and two cases of RO defined two common regions, namely a 1.5 Mb region within 10q11.23-q21.1 (ACF, D45864, PRKG1 and CSTF2T) and a 2.5 Mb region within 10q21.1 (ZWINT). Evaluating the global gene expression of distinct types of renal cell tumors, we did not find correlation between loss of the chromosome 10q regions and expression of the genes mentioned above. Loss at chromosome 11p15.2-p12 including a loss of 1.2 Mb region (SOX6) was found in two ROs and a chromophobe RCC. Partial losses of chromosome 12 (p13.33-p12.1) were seen in three chRCCs and gain at this region in one RO.

**Figure 4:**
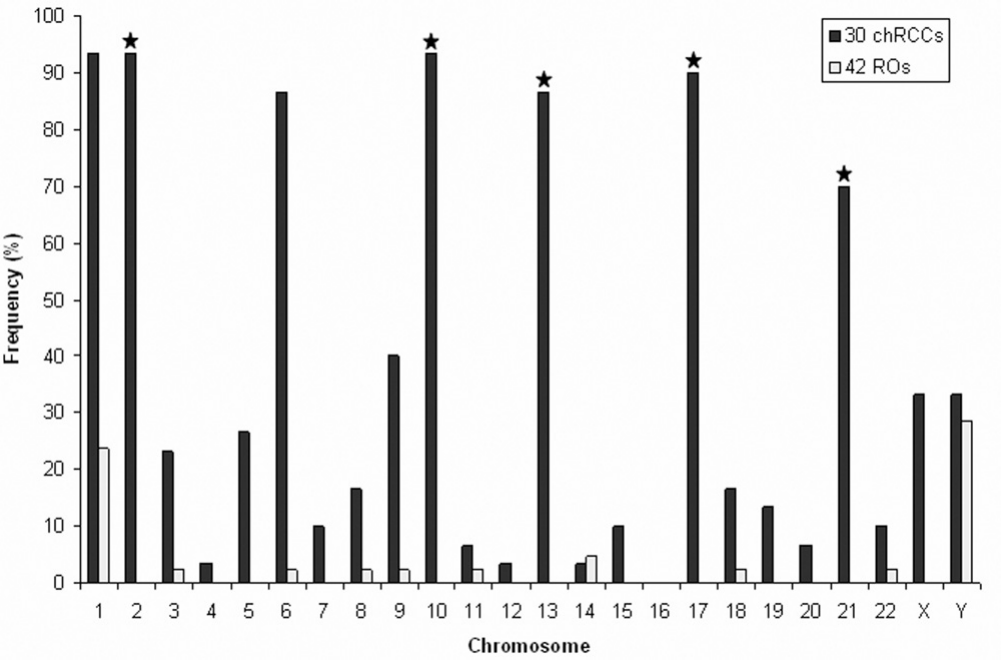
**Frequency of loss of entire chromosomes in chRCCs and ROs**. Chromosomal changes occurring exclusively in chromophobe RCCs are marked by star.

Comparing the genetic changes in 30 chromophobe RCCs and 42 ROs, we found some highly discriminating alterations. Loss of chromosome 2, 10, 13, 17 and 21 occurred in 93%, 93%, 87%, 90% and 70% of chromophobe RCCs, respectively. None of the 42 ROs displayed loss of these chromosomes (Figure 4).

### Comparison of gene expression level and copy number changes

**Figure 5:**
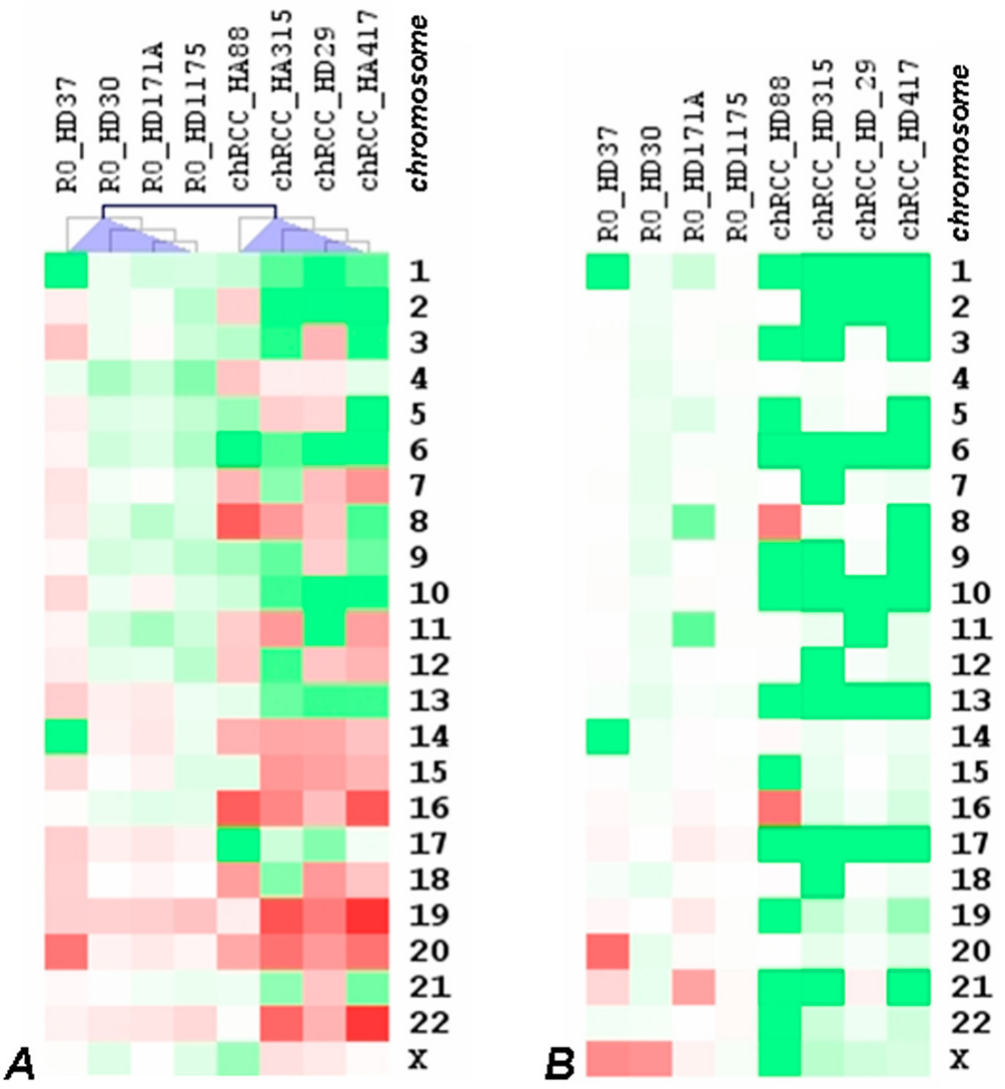
**Hierarchical clustering of gene expression profiles and comparative genomic microarray analysis of RO and chRCC**. (A) Genomic regions showing a significant number of up- and down-regulated genes are red and green, respectively. (B) Copy number changes of chromophobe RCCs and ROs are marked by green (loss) and red (gain). Generally, loss of chromosomes 1, 2, 6, 10, 13, 17 and 21 is associated with the downregulation of genes. However, genes at chromosomes 16, 19, 20 and 22 found to be overexpressed in spite of the normal diploid copy number of these chromosomes.

To evaluate the impact of specific chromosomal losses on gene expression, we analysed the gene expression data of the four chromophobe RCCs and four ROs that have also been analysed by the SNP array. First, to identify and summarize the regional (chromosomal) expression biases we segregated individual gene expression values into sets based on entire chromosome mapping. For each set, expression values from multiple probes that map within a given chromosome were condensed by averaging. Finally, gene expression profiles in four chromophobe RCCs and four ROs were organized by hierarchical clustering (Figure 5A). The chromosomal profiles correspond well with chromosomal losses obtained by the SNP array study for these chRCCs (Figure 5B). Namely, frequent downward expression biases were identified for chromosomes 1, 2, 3, 6, 10 and 13, which are commonly lost from the genome of chRCC. In the RO HD37 monosomie of chromosomes 1 and 14 were accompanied by decreased expression of genes from these two chromosomes. Similarly, monosomies of chromosomes 7, 9, 12 and 18 in the chromophobe RCC HA315 was associated with decreased expression of genes localized to these chromosomes. Chromosomes 8 and 16 were duplicated in the chromophobe RCC HD88 and corresponding to the three copies, genes were over-expressed from these chromosomes. Additionally, this approach predicted frequent over-expression of genes localized to chromosomes 14, 15, 16, 19, 20 and 22 in chRCC and to chromosome 19 in RO. Because several hundred genes are affected by the copy number and expression changes, it is impossible to identify specific genes, which may be involved in the genetics of chromophobe RCC or ROs.

## Discussion

We have applied high-density SNP-oligoarray to detect copy number alterations at specific chromosomal regions in chromophobe RCCs and ROs, which may mark the loci of genes involved in tumor development. Although we have detected genetic changes at several small regions of 10–100 kb in size, no recurrent alterations at these regions have been found in the tumors. As the high resolution SNP array is suitable to detect DNA alterations in a range of 10–30 kb, we can exclude the presence of recurrent small interstitial deletions in chromophobe RCCs and ROs with all certainty. The small random "losses" or "gains" may result from mitotic recombination events and probably do not play a role in the biology of tumor cells at all. Detection of such random genetic events in tumor cells by LOH studies may be misleading, as in our study, when searching for tumor suppressor genes [26].

The gain of a small DNA segment around exon 5 of the FHIT gene in three ROs from the same patient resulted also from the plasticity of genome, in this case from the instability of the most common fragile site FRA3B, rather than a positive selection of gene alteration responsible for tumor development

Although our genome wide SNP-array analysis failed to identify tumor suppressor gene loci, we have confirmed the high specificity of losses of chromosomes 1, 2, 6, 10, 13, 17 and 21 in chromophobe RCCs as it was shown by classical karyotyping, chromosomal CGH and microsatellite allelotyping several years ago [8,13,16,27]. Our study also revealed other chromosomal losses including monosomy of chromosome 3, 5 and 9 in 23–40% of the cases. Comparing the global gene expression to the expected DNA copy number changes in conventional, papillary and chromophobe RCCs, a frequent downward biases has been identified for chromosomes 1, 2, 6, 10q, and 17q in chromophobe RCCs [18]. We also found a decreased expression of genes from the chromosomes showing monosomies and also an increased expression of genes from chromosomes showing increased copy number in both chromophobe RCCs and ROs.

Which of the genes down-regulated by multiplex chromosomal losses in chromophobe RCCs or ROs are responsible for the tumor development and progression remains from these studies unknown. Loss of chromosome 17, which occurs in 90% of chromophobe RCCs, is associated with p53 tumor suppressor gene mutations in only 27% of the cases [28]. In spite of the frequent loss (86%) of chromosome 10, no mutation of the PTEN tumor suppressor gene has been found in chromophobe RCCs [29]. The germ line mutation of the folliculin gene is associated with the BHD syndrome and the development of so-called "mixed" chromophobe-oncocytic renal cell tumors [30]. However, the folliculin gene has been excluded to be instrumental in the development of sporadic chromophobe RCCs and ROs [31].

During preparation of this manuscript, a paper on the combined DNA and RNA analysis of chromophobe RCCs and ROs has been published [32]. They found an over-expression of genes from chromosome 19, especially the specific over-expression of ELGN2 in renal oncocytomas. We confirmed the increased transcriptional activity at 19 in both types of tumors, but also found frequent over-expression of genes along chromosomes 14, 15, 16, 20 and 22 in chromophobe RCCs. We found an elevated expression the ELGN2 in chromophobe RCCs and ROs, and also in some conventional and papillary RCCs as well. There are some discrepancies between the results regarding the DNA copy number changes. They found "an amplification of the entirety of chromosome 19 in chromophobe RCCs" and presented a slightly elevated signal at chromosome 19 in all the three tumors in Figure 1C. In contrary, we did not find any gain but the loss of chromosome 19 in four of the 30 chromophobe RCCs in our series. They also showed a gain at chromosome 7 in all the three chromophobe RCCs, the alteration that occurred only in one of the 30 chromophobe RCCs in our series.

Until now, the global gene expression of over 300 renal tumors including about 24 chromophobe RCCs and 20 renal oncocytomas in different series have been studied by filter and microarray hybridization. Most of these studies separated the group of chRCC/RO from other types of renal cancers by expression profile, but did not distinct chromophobe RCCs from ROs [19-21,23]. Some randomly selected antibodies were used to characterize distinct types of renal tumors, including chRCCs and ROs, but with controversial results. Markers such as cytokeratin 7, parvalbumin or claudin 7 were found to be expressed in the majority of chromophobe RCCs, but rarely in renal oncocytomas [20,23,33-35]. It was suggested that the kidney-specific cadherin is specifically expressed in chromophobe RCC, but others challenged their results [36,37]. Recently, MAL2 protein was found to be preferentially expressed in chromophobe RCCs, paralleling its mRNA differential expression according to the array analysis [22]. However, its expression was seen in one of five RO cases tested, and therefore the clinical diagnostic usefulness of this marker needs to be further validated by additional large-scale studies. Summing up the data from the literature, only spare and not specific data are available on the expression signature of chromophobe RCCs and renal oncocytomas and no reliable molecular targets have been identified in any examinations for the critical differential diagnosis of chromophobe RCC versus oncocytoma. Generally, 100% diagnostic tumor classification and discrimination cannot be based on a single gene product. Only a panel of marker genes could result in additive diagnostic reliability and would increase discriminative efficiency.

## Conclusion

In conclusion, in this first report on the high resolution DNA-array analysis on a large number of chromophobe RCCs and ROs we have excluded with all certainty the occurrence of small specific deletions. These types of renal cell tumors are characterised by the monosomies of specific chromosomes. Some of the genetic changes may occur in both types of tumors but the loss of entire chromosomes 2, 10, 13, 17 and 21 occurs exclusively in chromophobe RCCs. We have detected the loss of at least three of them in addition to the loss of chromosome 1 in each case analysed. Based on our results, any microsatellites or BAC clones localised at these chromosomes can be used to establish the diagnosis of chromophobe RCCs in cases with uncertain histology. This can be achieved in most histopathological laboratories by applying microsatellite analysis or FISH to detect the specific genetic alterations.

## Competing interests

The authors declare that they have no competing interests.

## Authors' contributions

MVY carried out the SNP experiments, the RT-PCR studies and evaluated the Affymetrix array. RPK and AGK supervised the SNP experiments. TB and BL collected the material, supplied the clinical data. GK have designed, coordinated and supervised the study. All authors read and approved the final manuscript.

## Pre-publication history

The pre-publication history for this paper can be accessed here:

http://www.biomedcentral.com/1471-2407/9/152/prepub

## References

[B1] Akhtar M Kfoury H Kardar A Linjawi T Kovacs G Sarcomatoid chromophobe cell carcinoma of the kidney J Urol Pathol 1996 4 155 166

[B2] Badoual C Tissier F Lagorce-Pages C Delcourt A Vieillefond A Pulmonary metastases from a chromophobe renal cell carcinoma: 10 years' evolution Histopathology 2002 40 300 302 10.1046/j.1365-2559.2002.1363e.x 11895501

[B3] Crotty TB Farrow GM Lieber MM Chromophobe cell renal carcinoma: clinicopathological feature of 50 cases J Urol 1995 154 964 967 10.1016/S0022-5347(01)66944-1 7637102

[B4] Renshaw AA Henske EP Loughlin KR Shapiro C Weinberg DS Aggressive variants of chromophobe renal cell carcinoma Cancer 1996 78 1756 1761 10.1002/(SICI)1097-0142(19961015)78:8<1756::AID-CNCR16>3.0.CO;2-X 8859189

[B5] Davis CJ Sesterhenn IA Mostofi FK Ho CK Renal oncocytoma. Clinicopathological study of 166 patients J Urogenital Pathol 1991 1 41 52

[B6] Kovacs G Soudah B Hoene E Binucleated cells in a human renal cell carcinoma with 34 chromosomes Cancer Genet Cytogenet 1988 31 211 215 10.1016/0165-4608(88)90219-1 3349439

[B7] Kovacs A Kovacs G Low chromosome number in chromophobe renal cell carcinomas Genes Chromosomes Cancer 1992 4 267 268 10.1002/gcc.2870040313 1382570

[B8] Speicher MR Schoell B du Manoir S Schröck E Ried T Cremer T Störkel S Kovacs A Kovacs G Specific loss of chromosomes 1, 2, 6, 10, 13, 17 and 21 in chromophobe renal cell carcinomas revealed by comparative genomic hybridisation Am J Pathol 1994 145 356 364 7519827 PMC1887405

[B9] Bugert P Gaul C Weber K Akhtar M Ljungberg B Kovacs G Specific genetic changes of diagnostic importance in chromophobe renal cell carcinomas Lab Invest 1997 76 203 208 9042156

[B10] Brunelli M Eble JN Zhang S Martignoni G Delahunt B Cheng L Eosinophilic and classic chromophobe renal cell carcinomas have similar frequent losses of multiple chromosomes from among chromosomes 1, 2, 6, 10, and 17, and this pattern of genetic abnormality is not present in renal oncocytoma Mod Pathol 2004 18 161 169 10.1038/modpathol.3800286 15467713

[B11] Füzesi L Gunawan B Braun S Boeckmann W Renal oncocytoma with a translocation t(9;11)(p23;q13) J Urol 2004 152 471 472 10.1016/s0022-5347(17)32766-0 8015093

[B12] Presti JC Moch H Reuter VE Huynh D Waldman FM Comparative genomic hybridisation for genetic analysis of renal oncocytomas Genes Chromosomes Cancer 1996 17 199 204 10.1002/(SICI)1098-2264(199612)17:4<199::AID-GCC1>3.0.CO;2-Z 8946201

[B13] Nagy A Buzogany I Kovacs G Microsatellite allelotyping differentiates chromophobe renal cell carcinomas from renal oncocytomas and identifies new genetic changes Histopathology 2004 44 542 546 10.1111/j.1365-2559.2004.01884.x 15186268

[B14] Herbers J Schullerus D Chudek J Bugert P Kanamaru H Zeisler J Ljungberg B Akhtar M Kovacs G Lack of genetic changes at specific genomic sites separates renal oncocytomas from renal cell carcinomas J Pathol 1998 184 58 62 10.1002/(SICI)1096-9896(199801)184:1<58::AID-PATH987>3.0.CO;2-1 9582528

[B15] Kovacs G Application of molecular cytogenetic techniques to the evaluation of renal parenchymal tumours J Cancer Res Clin Oncol 1990 116 318 323 10.1007/BF01612912 2202729 PMC12201232

[B16] Bugert P Kovacs G Molecular differential diagnosis of renal cell carcinomas by microsatellite analysis Am J Pathol 1996 149 2081 2088 8952540 PMC1865333

[B17] Wilhelm M Veltman JA Olshen A Jain AN Moore DH Presti JC Kovacs G Waldman FM Array based CGH for the differential diagnosis of renal cell cancer Cancer Res 2002 62 957 960 11861363

[B18] Furge KA Lucas KA Takahashi M Sugimura J Kort EJ Kanayama HO Kaqawa S Hoekstra P Curry J Yang XJ Teh BT Robust classification of renal cell carcinoma based on gene expression data and predicted cytogenetic profiles Cancer Res 2004 64 4117 4121 10.1158/0008-5472.CAN-04-0534 15205321

[B19] Takahashi M Yang XY Sugimura J Backdahl J Tretiakova M Qian CN Gray SG Knapp R Anema J Kahnoski R Nicol D Vogelzang NJ Furge KA Kanayama H Kagawa S The BT Molecular subclassification of kidney tumors and the discovery of new diagnostic markers Oncogene 2003 22 6810 6818 10.1038/sj.onc.1206869 14555994

[B20] Young AN Amin MB Moreno CS Lim SD Cohen C Petros JA Marshall FF Neish AS Expression profiling of renal epithelial neoplasms: a method for tumor classification and discovery of diagnostic molecular markers. Am J Pathol 2001 58 11639 11651 10.1016/S0002-9440(10)64120-X PMC1891957 11337362

[B21] Higgins JPT Shinghal R Gill H Reese JH Terris M Cohen RJ Fero M Pollack JR Rijn M van de Brooks JD Gene Expression Patterns in Renal Cell Carcinoma Assessed by Complementary DNA Microarray Am J Pathol 2003 162 925 932 12598325 10.1016/S0002-9440(10)63887-4 PMC1868114

[B22] Rohan S Tu JJ Kao J Mukherjee P Campagne F Zhou XK Hyjek E Alonso MA Chen YT Gene Expression Profiling Separates Chromophobe Renal Cell Carcinoma from Oncocytoma and Identifies Vesicular Transport and Cell Junction Proteins as Differentially Expressed Genes Clin Cancer Res 2006 12 6937 6945 10.1158/1078-0432.CCR-06-1268 17145811

[B23] Schuetz AN Yin-Goen Q Amin MB Moreno CS Cohen C Hornsby CD Yang WL Petros JA Issa MM Pattaras JG Oqan K Marshall FF Young AN Molecular classification of renal tumors by gene expression profiling J Mol Diagn 2005 7 206 218 15858144 10.1016/S1525-1578(10)60547-8 PMC1867531

[B24] Kovacs G Akhtar M Beckwith BJ Bugert P Cooper CS Delahunt B Eble JN Fleming S Ljungberg B Medeiros KJ Moch H Reuter VE Ritz E Roos G Schmidt D Srigley JR Störkel S Berg E van den Zbar B The Heidelberg classification of renal cell tumours J Pathol 1997 183 131 133 10.1002/(SICI)1096-9896(199710)183:2<131::AID-PATH931>3.0.CO;2-G 9390023

[B25] Nannya Y Sanada M Nakazaki K Hosoya N Wang L Hangaishi A Kurokawa M Chiba S Bailey DK Kennedy GC Ogawa S A robust algorithm for copy number detection using high-density oligonucleotide single nucleotide polymorphism genotyping arrays Cancer Res 2005 65 6071 6079 10.1158/0008-5472.CAN-05-0465 16024607

[B26] Tischfield YA Loss of heterozygsity or: How I learned to stop worrying and love mitotic recombination Am J Hum Genet 1997 61 995 999 9345110 10.1086/301617 PMC1716040

[B27] Kovacs G Molecular differential pathology of renal cell tumours Histopathology 1993 22 1 8 10.1111/j.1365-2559.1993.tb00061.x 8436337

[B28] Contractor H Zariwala M Bugert P Zeisler J Kovacs G Mutation of the p53 tumour suppressor gene occurs preferentially in chromophobe type of renal cell tumours J Pathol 1997 181 136 139 10.1002/(SICI)1096-9896(199702)181:2<136::AID-PATH766>3.0.CO;2-2 9120715

[B29] Sükösd F Digon B Fischer J Pietsch T Kovacs G Allelic loss at chromosome 10q23.3 but lack of mutation of the PTEN/MMAC1 in chromophobe renal cell carcinoma Cancer Genet Cytogenet 2001 128 161 163 10.1016/S0165-4608(01)00413-7 11463457

[B30] Pavlovich CP Walther MM Eyler RA Hewitt SM Zbar B Linehan WM Merino MJ Renal tumours in Birt-Hogg-Dube syndrome Am J Surg Pathol 2002 26 1542 1552 10.1097/00000478-200212000-00002 12459621

[B31] Nagy A Zubakov D Stupar Z Kovacs G Lack of mutation of the folliculin gene in sporadic chromophobe RCC and renal oncocytoma Int J Cancer 2004 109 472 475 10.1002/ijc.11694 14961590

[B32] Koeman JM Russell RC Tan MH Petillo D Westphal M Koelzer K Metcalf JL Zhang Z Matsuda D Dykema KJ Houseman HL Kort EJ Furge LL Kahnoski RJ Richard S Vieillefond A Swiatek PJ The BP Ohh M Furge KA Somatic pairing of chromosome 19 in renal oncocytoma is associated with deregulated ELGN2-mediated oxygen-sensing response PLoS Genet 2004 4 e1000176 10.1371/journal.pgen.1000176 PMC2518213 18773095

[B33] Wu SL Kothari P Wheeler TM Reese T Connelly JH Cytokeratins 7 and 20 immunoreactivity in chromophobe renal cell carcinomas and renal oncocytomas Mod Pathol 2002 15 712 717 10.1097/01.MP.0000017566.29755.8A 12118108

[B34] Abrahams NA MacLennan GT Khoury JD Ormsby AH Tamboli P Doglioni C Schumacher B Tickoo SK Chromophobe renal cell carcinoma: a comparative study of histological, immunhistochemical and ultrastructural features using high throughput tissue microarray Histopathology 2004 45 593 602 10.1111/j.1365-2559.2004.02003.x 15569050

[B35] Young AN de Oliveira Salles PG Lim SD Cohen C Petros JA Marshall FF Neish AS Amin MB Beta defensin-1, parvalbumin, and vimentin: A panel of diagnostic immunohistochemical markers for renal tumours derived from gene expression profiling studies using cDNA microarrays Am J Surg Pathol 2005 27 199 205 10.1097/00000478-200302000-00008 12548166

[B36] Mazal PR Exner M Haitel A Kreiger S Thomson RB Aronson PS Susani M Expression of kidney specific cadherin distinguishes chromophobe renal cell carcinoma from renal oncocytoma Hum Pathol 2005 36 22 28 10.1016/j.humpath.2004.09.011 15712178

[B37] Chen YT Tu JJ Kao J Zhou XK Mazumdar M Messenger RNA expression ratios among four genes predict subtypes of renal cell carcinoma and distinguish oncocytoma from carcinoma Clin Cancer Res 2005 11 6558 6566 10.1158/1078-0432.CCR-05-0647 16166433

